# Clinical significance of distal femur morphology in a healthy Mongolian youth population

**DOI:** 10.1038/s41598-023-35463-3

**Published:** 2023-05-20

**Authors:** Boyang Wang, Guoliang Zhang, Ribusurong Pu, Qiang Li, Yuewen Wang

**Affiliations:** 1 Department of Orthopedics, The Affiliated Hospital of Inner Mongolia Medical University, Tongdaobeilu No.1, Hohhot, 010050 Inner Mongolia Autonomous Region China; 2 Musculoskeletal Tumor Center, Peking University People’s Hospital, Beijing, China; 3 Beijing Key Laboratory of Musculoskeletal Tumor, Beijing, China

**Keywords:** Musculoskeletal system, Image processing

## Abstract

Morphological parameters of knee joint are related to race and nationality. At present, knee prosthesis come from white male population. Due to the mismatch between the prosthesis and other ethnic groups, the prosthesis life span is reduced, revision surgery and the patients’ economic burden are increased. There is no data of the Mongolian ethnic group. In order to treat patients more accurately, we measured the Mongolian data of the femoral condyle. A total of 122 knee joints were scanned in 61 volunteers (21 males and 40 females) with an average age of 23.259 ± 1.395 years. The Mimics software was used to reconstruct the 3D image and measure the data of each line. The data were analyzed by statistical methods such as t test, and P < 0.05 was taken as the significant. 122 normal femoral condyle data were obtained. The mean transverse diameter of femoral condyle is 76.472 ± 5.952 mm, medial condyle is 29.259 ± 11.461 mm, and the sagittal diameter of the medial condyle was 56.758 ± 4.163 mm. The transverse diameter of the lateral femoral condyle is 29.388 ± 3.157 mm, the sagittal diameter of the lateral condyle is 58.937 ± 3.527 mm and the femoral plane rate is 1.264 ± 0.072. (1) There was no statistical significance in the left and right knee joint data (P > 0.05). (2) The different genders data of femoral condyle were statistically significant (P < 0.05). (3) Compared with other nationalities and races, the data of femoral condyle are different. (4) There are differences between femoral surface ratio and mainstream prosthesis data.

## Introduction

Total knee arthroplasty (TKA) plays an important role in the treatment of rheumatoid arthritis of the knee, severe osteoarthritis of the knee, and knee deformities. In particular, TKA is the only effective treatment option for advanced knee diseases that severely affect patients’ daily lives and work ability. Most knee prostheses currently used in clinical practice in China are designed and manufactured using the measured parameters of the knee joint in Caucasians. Existing studies indicate that the morphological parameters of the knee joint are closely related to race and ethnicity^1,2^. The sagittal diameter of the femoral condyle of the knee is larger in Asians than in Caucasians, resulting in a sagittal to transverse diameter ratio that does not match that of mainstream knee prostheses. This leads to a shorter life span of the prosthesis and increased risk of revision surgery for and financial burden on the patient^3–9^. There are reports on the femoral condyle morphology in the Han, Hui, Uyghur, and Zhuang populations, but no studies to date have documented the geometry of the normal femoral condyle in the Mongolian population. In this study, we measured and analyzed the geometry of the femoral condyle in healthy Mongolian adults in the Inner Mongolia Autonomous Region, measured the transverse diameter of the femoral condyle, the transverse and sagittal diameters of the inner and outer condyle, and the mediolateral/lateral anteroposterior (ML/LAP) ratio of the femoral surface ratio and analyzed each parameter in the hope that the geometry of the femoral condyle of Mongolian people can be used to design a suitable knee prosthesis for Chinese people. We hope that this information will contribute to more suitable knee prostheses for Mongolian people.

## Material and methods

### Ethics statement

This study was carried out in accordance with the approval by the ‘The Affiliated Hospital of Inner Mongolia Medical University’ with written informed consent from all subjects. All subjects provided written informed consent in accordance with the Declaration of Helsinki.

### General data

Healthy Mongolian adults living in Hohhot, Inner Mongolia Autonomous Region, were recruited from September to December 2010. Our local ethics committee approved this study, and all volunteers provided written informed consent. A total of 61 volunteers (21 men, 40 women) aged 20–27 years (mean, 23.295 ± 1.395 years) were enrolled. Inclusion criteria were: (1) indigenous to Inner Mongolia Autonomous Region, no history of inter-ethnic marriage within three generations, and not currently pregnant or lactating; (2) no obvious knee deformity, no bone destruction of the knee joint, and no alteration of the knee gap; (3) no discomfort such as swelling or pain in either knee or motion limitations in daily life, no other systemic diseases, no history of knee trauma or disease, and denial of a history of knee surgery; and (4) willing and able to undergo a computed tomography (CT) examination.

### CT and three-dimensional reconstruction

The volunteers were placed in a supine position with the lower extremities flat and both knees straight in a neutral position, and the CT (GE Discovery HD) scan was performed of the knee gap, including the distal 1/3 of the femur and the proximal 1/3 of the tibia; the designed scan layer thickness was 0.625 mm and layer spacing was 0.5 mm, a bone filter and a soft-tissue filter were used^10^. Three-dimensional reconstruction of the imaged areas was performed and the parameters of the reconstructed femoral condyle were measured using Mimics 16.0.

### Definition and measurement of parameters related to the femoral condyle

A total of three orthopedic doctors (chief physicians) participated in the measurement of CT images, and they jointly decided the starting point and end point of the morphological parameters of the femoral condyle.

The femoral condylar parameters measured in this study included: mediolateral (ML), lateral condyle (LC), lateral anteroposterior (LAP), femoral mediolateral condyle (FMLC), femoral medial anteroposterior (FMAP), and ML/LAP.

**Figure 1:**
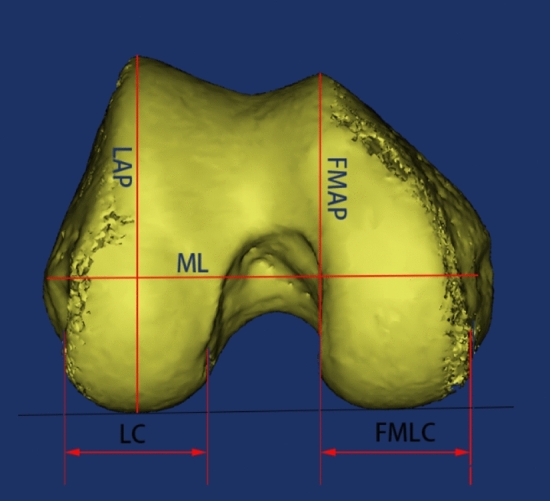
The image is a three-dimensional schematic of the femoral condyle reconstructed by Mimics software. The femoral condylar parameters measured in this image included: mediolateral (ML), lateral condyle (LC), lateral anteroposterior (LAP), femoral mediolateral condyle (FMLC), femoral medial anteroposterior (FMAP), and ML/LAP.

The relevant parameters are defined as follows (Fig. 1). ML, the line between the most concave point of the medial epicondyle and the most convex point of the lateral epicondyle of the femoral condyle, is used clinically as a marker of the rotational alignment of the distal femur. LC is the distance between the plumb line made across each of the lateral and medial edges of the lateral condyle. LAP is a plumb line over the highest point of the lateral anterior condyle for the posterior condylar line. FMLC is the distance between the plumb lines across each of the lateral and medial edges of the medial condyle. FMAP is a plumb line of the posterior condyle line past the highest point of the medial anterior condyle.

### Statistical processing

To clarify the adequacy of the sample size, a post hoc power analysis was performed, using G power 3.1. The alpha value was 0.05, sample size is 61 and effect size was 0.8.

The measured data were recorded using Excel software (accurate to three decimal and corrected by rounding), and the mean ± standard deviation of each parameter was calculated separately using SPSS 26 statistical software. The data were judged to be normally or non-normally distributed. Normally distributed data were examined using the t-test to determine whether they were statistically significant, while non-normally distributed data were examined using the Wilcoxon signed-rank test. Values of P < 0.05 were considered statistically significant.

## Results

According to the calculation in G power 3.1, the power is equal to 0.9999858. The sample size is adequacy.

The statistical analysis revealed that the values were not statistically different between the left and right sides of the groups (P > 0.05; Table 1); thus, they were combined. We found differences in the transverse diameters of the femoral condyle, transverse and sagittal diameters of the femoral medial condyle, transverse and sagittal diameters of the femoral epicondyle, and ML/LAP between the sexes, with men having significantly greater values than women (P < 0.05; Table 2). By comparing the knee joint morphology of different genders in Mongolia, it can be seen that the male knee joint is more flat than the female, and the results are similar to those of other races^11^.

**Table 1:** Volunteers left and right knee joints statistical results.

		R	L	P
ML	Men	82.884 ± 4.585	79.769 ± 12.498	0.255
	Women	73.197 ± 2.838	73.152 ± 3.581	0.896
FMLC	Men	32.555 ± 11.368	36.385 ± 18.530	0.274
	Women	26.792 ± 7.617	26.253 ± 7.649	0.055
FMAP	Men	60.414 ± 4.430	59.481 ± 4.741	0.181
	Women	55.237 ± 2.657	54.930 ± 2.852	0.472
LC	Men	31.674 ± 3.255	31.042 ± 3.939	0.487
	Women	28.290 ± 2.593	28.418 ± 2.079	0.642
LAP	Men	63.919 ± 3.390	63.366 ± 4.080	0.314
	Women	58.642 ± 2.666	59.231 ± 4.233	0.269
MP/LAP	Men	1.297 ± 0.045	1.265 ± 0.208	0.461
	Women	1.230 ± 0.055	1.239 ± 0.080	0.289

*R* right knee, *L* lift knee.

**Table 2:** Statistical results of Mongolian male and female femur condyle data.

	Men	Women	Total	t	P
ML	82.755 ± 4.799	73.174 ± 3.210	76.472 ± 5.952	11.642	0.000
FMLC	34.470 ± 15.306	26.523 ± 7.589	29.259 ± 11.461	20.489	0.003
FMAP	59.948 ± 4.556	55.084 ± 2.743	56.758 ± 4.163	20.489	0.000
LC	31.358 ± 3.583	28.354 ± 2.336	29.388 ± 3.157	49.140	0.000
LAP	60.557 ± 4.224	63.643 ± 3.716	58.937 ± 3.527	6.847	0.000
MP/LAP	1.302 ± 0.065	1.244 ± 0.068	1.264 ± 0.072	0.866	0.000

## Discussion

With improvements in quality of life, changes in lifestyle habits, and increases in average patient age, the incidence and detection rate of knee disorders are increasing annually and the number of Chinese people receiving TKA has increased significantly. TKA is a major advancement in the development of orthopedic science in the last half century with a wide range of indications, and it has become an important way to improve knee function and improve the quality of life for patients with osteoarthritis, rheumatoid arthritis, severe knee trauma, and other serious knee diseases. Its postoperative results are good and the long-term follow-up results are satisfactory. However, 20% of patients with TKA are still unsatisfied after surgery^12^, and postoperative complications are particularly worrying, which has attracted widespread attention from orthopedic surgeons. The postoperative results, long-term outcomes, and incidence of postoperative complications after TKA are closely related to knee prosthesis design, intraoperative osteotomy volume, osteotomy angle, soft tissue balance, and prosthesis placement level. Importantly, the data used in prosthesis design are the most influential in achieving a match in prosthesis geometry and dimensions and the osteotomy surface of the patient undergoing surgery, i.e., it is crucial that the intraoperatively applied prosthesis fits the patient’s knee geometry. It is well known that the placed knee prosthesis can lead to accurate knee joint alignment and significant changes in the biomechanical and anatomical structure of the knee joint^13–15^. Therefore, the matching of the artificial joint prosthesis and the osteotomy surface of the femoral condyle is an important influential factor in determining TKA surgery success and long-term prosthesis patency.

With regard to the above issues, relevant studies have demonstrated that Chinese body size is shorter than and the anteroposterior and left–right diameters of the proximal tibia are smaller than those of Caucasians^16^; similarly, the same problem exists with the femoral condyle prosthesis. Due to this inter-ethnic difference, it is not uncommon for imported artificial joint prostheses to be poorly matched when applied to Chinese patients. This mismatch between the prosthesis and the osteotomy surface can lead to knee pain, limited popping, and motion after TKA, ultimately affecting the knee’s normal function^17^. Such morphological data of the knee joint vary across ethnic groups worldwide^18^. Therefore, it is necessary to study the geometry of the femoral condyles of the knee joint in normal Mongolians and assess the differences between them and other Western ethnic and racial groups.

The analysis of this group of data revealed inter-sex differences in the geometry of the femoral condyle in Mongolians. All parameters of the femoral condyle, including the ML/LAP, were significantly greater in men than in women^11,19^. This indicates that the sagittal diameter of the knee condyle was larger in women than in men when the transverse diameter of the femoral condyle was the same; in the case of the same sagittal diameter, the transverse diameter of the femoral condyle was smaller in women than in men, which can lead to a tendency of the osteotomy surface to hang past the femoral condyle when a knee prosthesis is placed in the female knee if the sagittal match is used as the criterion for prosthesis selection, that is overhang^5,6,9^. This can lead to postoperative pressure and irritation of the soft tissues around the knee, which can cause painful symptoms and, in severe cases, affect soft tissue balance^6,20,21^. If the prosthesis is selected by matching of the transverse diameter, excessive osteotomy of the posterior condyle will occur, which will easily lead to unequal internal extension and flexion gaps similar to the findings of Western scholars^3^.

It was once believed that the morphological differences were small between sexes of the same race despite the parameters of the knee condyle varying. Thus, as long as the morphological design of the prosthesis was reasonable, different models could be used for different individuals to ensure proper matching. This view is deficient. The current commercially available knee prosthesis is designed and fabricated based on the geometric parameters of the knee joint of Caucasian men, resulting in a high degree of their adaptation to men, and when used by women, the problem of mismatch is likely to occur. Therefore, to achieve better knee function and prolong prosthesis life, prosthesis design and application must be refined and sex-specific. Plaster et al.^22^. suggested that when women use a knee prosthesis designed based on the geometric parameters of the female knee, early function was superior to that of other non-female parameter-based prostheses. In Kim's^23^ article, is noted that there was significant difference in the incidence of overhang of femoral component between the gender-specific implant and the traditional implant, but in terms of range of motion, Hospital for Special Surgery knee score, radiographic result, patella tilt angle and displacement, no significant difference was observed between two groups. But in other article^5^ point that femoral component overhang of > 3 mm in at least one zone was associated with an almost twofold increased risk of knee pain more severe than occasional or mild at 2 years after surgery (odds ratio, 1.9; 95% confidence interval, 1.1 to 3.3), however, femoral component overhang was not a significant predictor of postoperative flexion. The majority of knee prostheses currently used in China are imported or copied from imported prostheses, which also suffer from the above problems.

Although the use of domestic joint prostheses has surpassed that of imported joint prostheses in recent years, most of the domestic joints are mainly imitations and the geometric parameters are still derived from the measurement data of Caucasian men in Europe and the United States. This has led to frequent mismatches in the intraoperative use of prostheses in the national population. According to Zhang’s findings^24^ ML/LAP ratio of the domestic mainstream imported prosthesis system was 1.04–1.16, much smaller than the measurement data of this group. According to the Tables 3, 4, it can be seen that the mainstream knee prosthesis is quite different from the normal Mongolian morphology, because the SD value is not provided in the reference, so statistical comparison cannot be made, but comparing the ML/LAP value can find that the difference is huge. The comparison of the measurement data of this group with those of European and American and several Asian ethnic groups also revealed that most of the parameters of this group were smaller than the European and American data, while some were similar to those of Japanese (Tables 5 and 6). The differences in the measurement data of femoral condyles between normal Mongolians and different ethnic groups were significant, which would probably cause difficulty choosing the intraoperative prosthesis type and affect the long-term results of the knee joint as well as the survival time of the prosthesis. As seen in the above table, the differences in distal femoral geometry between different ethnic groups and races were significant, while the differences in ML/LAP ratios between different ethnic groups were statistically significant by the abstract independent samples t-test. Because of the poor fit between this prosthesis and the national knee osteotomy surface as well as its insufficient coverage, the gap between the actual service life of the prosthesis and the patient’s expected life span is large and repair operations are bound to increase, which undoubtedly increases the economic burden of the national population and the pain of the secondary operation.

**Table 3:** Comparison of ML/LAP data for normal Mongolian male and mainstream prosthetics.

	Minimum	Maximum	Mean
Mongolian (male)	1.167	1.542	1.302
DePuy sigma PFC^24^	1.053	1.087	1.075
LINK GEMINI I^24^	1.053	1.163	1.075
United U1^24^	1.136	1.149	1.149
Zimmer Nexgen^24^	1.064	1.163	1.111
Wright Advane^24^	1.111	1.136	1.124

**Table 4:** Comparison of ML/LAP data for normal Mongolian female and mainstream prosthetics.

	Minimum	Maximum	Mean
Mongolian (female)	1.083	1.441	1.244
DePuy sigma PFC^24^	1.053	1.087	1.075
LINK GEMINI I^24^	1.053	1.163	1.075
United U1^24^	1.136	1.149	1.149
Zimmer Nexgen^24^	1.064	1.163	1.111
Wright Advane^24^	1.111	1.136	1.124

**Table 5:** Morphological data of femoral condyle of different races and genders.

	ML		LAD		ML/LAP	
	Men	Women	Men	Women	Men	Women
Chuang^25^	80.04 ± 3.98	71.77 ± 3.89	61.34 ± 3.67	56.04 ± 2.47	–	–
Mongolian	82.755 ± 4.799	73.174 ± 3.210	60.557 ± 4.224	63.643 ± 3.716	1.302 ± 0.065	1.244 ± 0.068
Japanese^11^	82.60 ± 3.38	73.40 ± 3.66	63.40 ± 2.91	58.9 ± 3.63	1.31 ± 0.06	1.25 ± 0.09
Korean^26^	81.50 ± 5.70	76.70 ± 3.71	59.00 ± 4.01	58.4 ± 3.10	1.19 ± 0.04	1.33 ± 0.03
French^10^	83.7 ± 5.8	76.2 ± 14.1	70.3 ± 5.3	66.3 ± 13.6	1.19 ± 0.08	1.33 ± 0.15
African American^27^	84.9 ± 4.7	75.8 ± 3.3	61.2 ± 3.6	57.4 ± 8.3	1.39 ± 0.07	1.38 ± 0.34
Caucasian^27^	85.9 ± 4.7	75.8 ± 3.3	61.2 ± 3.6	55.9 ± 3.3	1.41 ± 0.06	1.36 ± 0.06

**Table 6:** Statistics of normal femoral condyle morphology of Mongolian and different races and nationalities.

	ML		ALP		ML/LAP	
	Men	Women	Men	Women	Men	Women
Chuang^25^	0.015	0.021	0.010	0.000	–	–
Japanese^11^	0.836	0.679	0.000	0.000	0.498	0.635
Korean^26^	0.207	0.000	0.036	0.000	0.000	0.000
French^10^	0.316	0.000	0.000	0.085	0.000	0.000
African American^27^	0.019	0.000	0.324	0.000	0.000	0.001
Caucasian^27^	0.000	0.000	0.261	0.000	0.000	0.000

In conclusion, there is an urgent need to design and manufacture an artificial knee prosthesis system that is suitable for national use. If combined with adequate preoperative surgical design and preparation, China’s artificial knee arthroplasty technology will achieve further needed improvements.

## Conclusion

It was found that there were statistical differences in the shape of femoral condyle in different ethnic groups. It indicates that the current mainstream knee prosthesis is not well matched with the Mongolian population, which leads to the reduction of the prosthesis life and the increase of revision probability. With the development of economy, people's demand for quality of life is becoming higher and higher. It is urgent to design an artificial knee prostheses system suitable for Chinese people.

## Data Availability

The datasets generated and analyzed during the current study are not publicly available due the raw data contains unpublished content, but are available from the corresponding author on reasonable request.
